# Hypothalamic miRNAs: emerging roles in energy balance control

**DOI:** 10.3389/fnins.2015.00041

**Published:** 2015-02-12

**Authors:** Marc Schneeberger, Alicia G. Gomez-Valadés, Sara Ramirez, Ramon Gomis, Marc Claret

**Affiliations:** ^1^Diabetes and Obesity Research Laboratory, Institut d'Investigacions Biomèdiques August Pi i SunyerBarcelona, Spain; ^2^Department of Endocrinology and Nutrition, School of Medicine, Hospital Clínic, University of BarcelonaBarcelona, Spain; ^3^CIBER de Diabetes y Enfermedades Metabólicas AsociadasBarcelona, Spain

**Keywords:** hypothalamus, miRNA, POMC neurons, obesity, energy balance, mouse models

## Abstract

The hypothalamus is a crucial central nervous system area controlling appetite, body weight and metabolism. It consists in multiple neuronal types that sense, integrate and generate appropriate responses to hormonal and nutritional signals partly by fine-tuning the expression of specific batteries of genes. However, the mechanisms regulating these neuronal gene programmes in physiology and pathophysiology are not completely understood. MicroRNAs (miRNAs) are key regulators of gene expression that recently emerged as pivotal modulators of systemic metabolism. In this article we will review current evidence indicating that miRNAs in hypothalamic neurons are also implicated in appetite and whole-body energy balance control.

## Obesity and the hypothalamic regulation of energy balance

The obesity epidemic has become a global issue (Finucane et al., 2011), causing major human and economic consequences. Obesity is associated to high rates of morbidity and mortality due to a major risk to develop serious and chronic conditions, such as type-2 diabetes, cardiovascular diseases, hypertension, stroke, and certain forms of cancer (Kopelman, 2000). However, and despite the magnitude of this public health problem, effective and safe pharmacological strategies are currently unavailable (Dietrich and Horvath, 2012). Consequently, understanding the precise mechanisms implicated in energy balance control is crucial for the development of more effective anti-obesity therapeutical approaches.

In recent years, the hypothalamus has firmly emerged as a critical area of the central nervous system (CNS) implicated in the regulation of whole-body energy homeostasis (Schneeberger et al., 2014). Specific subpopulations of neurons of the arcuate nucleus of the hypothalamus (ARC), such as neurons co-expressing orexigenic neuropeptides agouti-related protein (AgRP) and neuropeptide Y (NPY) and neurons co-expressing anorexigenic neuropeptides pro-opiomelanocortin (POMC) precursor and cocaine and amphetamine-related transcript (CART), play critical roles in energy homeostasis control (Schneeberger et al., 2014). These subsets of hypothalamic neurons (hereafter referred as AgRP and POMC neurons, respectively) are able to sense a wide variety of hormones and nutrient-related signals informing about the energy status of the organism. Under normal physiological conditions, the integration of these inputs results in appropriate and coordinated responses aimed at regulating energy balance through the modulation of appetite and energy expenditure (Schneeberger et al., 2014). Adequate responses to hormonal and nutritional signals by relevant neuronal subsets are achieved through the modulation of intracellular signaling pathways, which may culminate in the transcriptional regulation of specific genes.

## Biogenesis and functions of microRNAs

The precise molecular mechanisms by which POMC and AgRP neurons regulate particular gene expression programs are largely unknown. MicroRNAs comprise a highly conserved class of short (20–23 nucleotides) non-coding RNAs that represent an additional layer of complexity to the gene regulation process at post-transcriptional level (Krol et al., 2010). The current view suggest that a primary role for miRNAs is to confer robustness to those biological processes under constant perturbations by reinforcing transcriptional programs and attenuating aberrant transcripts (Ebert and Sharp, 2012). In this regard, miRNAs are excellent candidates to reliably control gene expression patterns aimed at generating appropriate physiological adaptive responses in hypothalamic neural circuits constantly exposed to hormonal and nutritional fluctuations.

**Figure 1:**
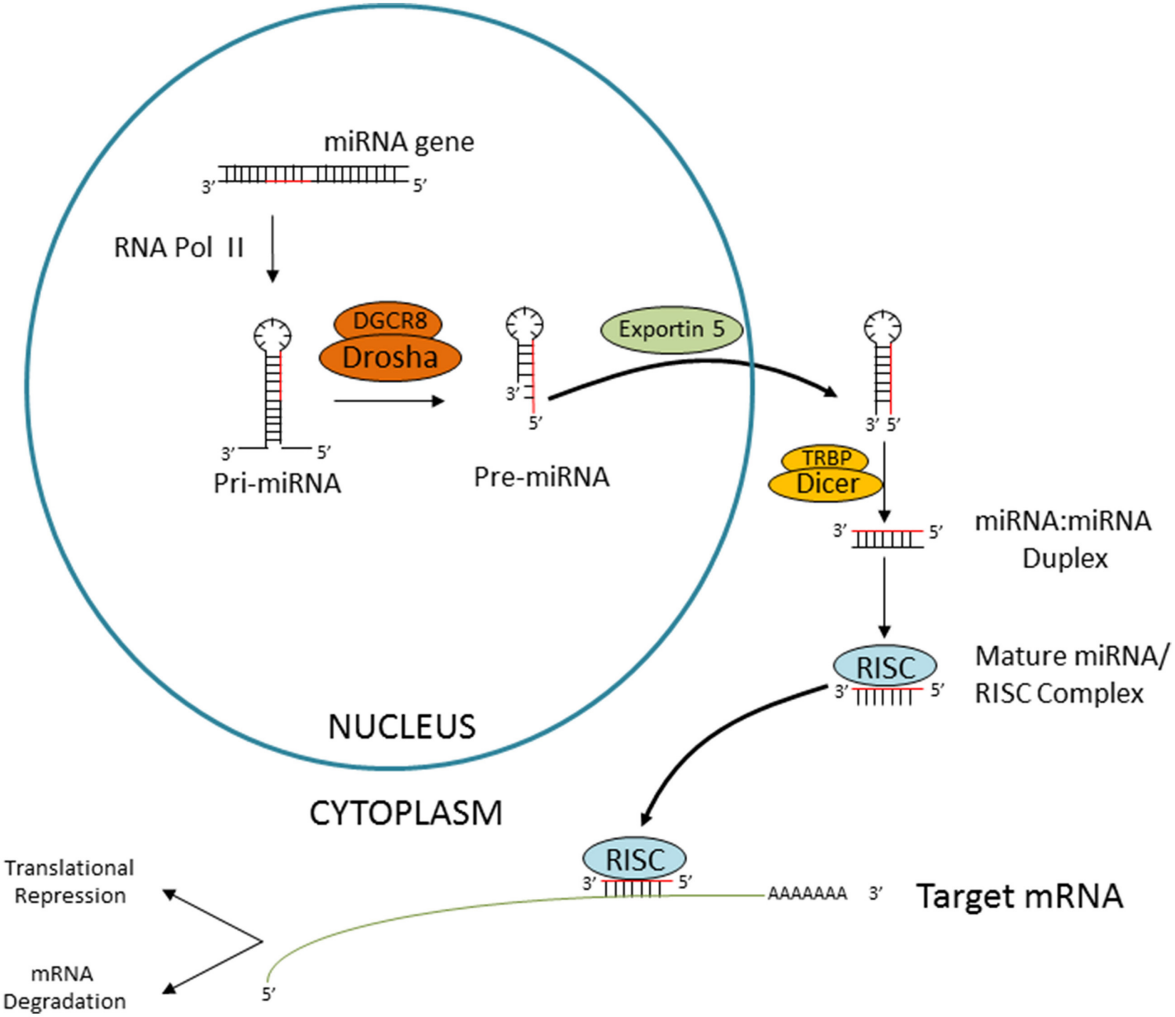
**Canonical miRNA biogenesis**. Schematic representation of the Dicer-dependent miRNA generation pathway. miRNA genes are transcribed by RNA polymerase II, followed by nuclear processing by the microprocessor complex (consitituted by DGCR8 and Drosha), cytoplasmatic export by Exportin 5 and Dicer-mediated processing. The miRNA duplex is then released and loaded into the RISC. The “passenger” strand is degraded and the so-called “guide” miRNA will interact with the target mRNA causing translational repression or degradation.

The majority of miRNAs are generated through a canonical process (Figure 1) (Ha and Kim, 2014). miRNA genes are transcribed by RNA polymerase II as long primary transcripts which contain a hairpin structure where miRNA sequences are encoded (pri-miRNA). Pri-miRNAs undergo nuclear processing by the Microprocessor complex (comprising Drosha and DGCR8), producing stem-loop precursors (pre-miRNAs). These pre-miRNAs will be exported to the cytoplasm and further processed by a complex containing the evolutionary conserved RNAse III-type endonuclease Dicer. Dicer cleaves the pre-miRNA terminal loop releasing a small RNA duplex, which is subsequently loaded into the RNA-induced silencing complex (RISC) (Ha and Kim, 2014). In general terms, miRNAs interact with the 3′ UTR region of target mRNAs causing its degradation and/or repression of protein translation (Figure 1) (Fabian et al., 2010).

Given the crucial regulatory role of miRNAs, it is not surprising that more than half of all mRNAs are estimated to be targets of miRNAs and each miRNA is predicted to regulate up to hundreds of targets. Consistent with this promiscuous activity, numerous studies indicate that aberrant miRNA function or expression can interfere with a range of biological processes and thus contribute to abnormal gene expression patterns associated with disease development, including neurological disorders, cardiovascular diseases, and cancer (Sayed and Abdellatif, 2011). Extensive evidence indicates that miRNAs also play key functions as regulators of metabolism in peripheral tissues such as liver, adipose tissue and pancreas (Fernandez-Valverde et al., 2011; McGregor and Choi, 2011; Moore et al., 2011; Rottiers and Naar, 2012). Remarkably, recent research has also started to reveal a key role for hypothalamic miRNAs upon systemic energy homeostasis.

## Hypothalamic miRNA processing machinery and its potential implication in the development of energy balance phenotypes

Components of the miRNA biogenesis machinery have been associated with energy homeostasis phenotypes. For example, transcriptomic analysis of the anorexia mouse model *anx/anx* revealed a tissue specific de-regulation of genes targeted by miRNAs (Mercader et al., 2012). In the hypothalamus, a preferential up-regulation of miRNA targets and miRNA-induced silencing complex genes (*Ago2, Pabpc1, Fmr1, Dgcr8*, and *Ddx6*) was found. Conversely, putative miRNA target genes tended to be downregulated in the cortex of *anx/anx* mice. To what extent the phenotype of the *anx/anx* mice (anorexia-caquexia, hyperactivity and ataxia) are cause or consequence of this differential miRNA regulation remains unknown, and requires further functional studies.

Dicer is necessary for the biogenesis of most miRNAs. Thus the characterization of conditional Dicer knock-out (KO) mice has been recently used as an initial step to evaluate the potential roles of miRNAs in a cell-specific manner. In the CNS, numerous cell and regional-specific Dicer KO mice have been generated demonstrating the importance of miRNAs in neuronal development, differentiation and survival (Meza-Sosa et al., 2014). Furthermore, temporal interventions also indicated developmental time-dependent functions for miRNAs. Defects in these biological processes results in serious CNS alterations, including behavioral alterations, depression, and neurodegerenation (Meza-Sosa et al., 2012).

**Figure 2:**
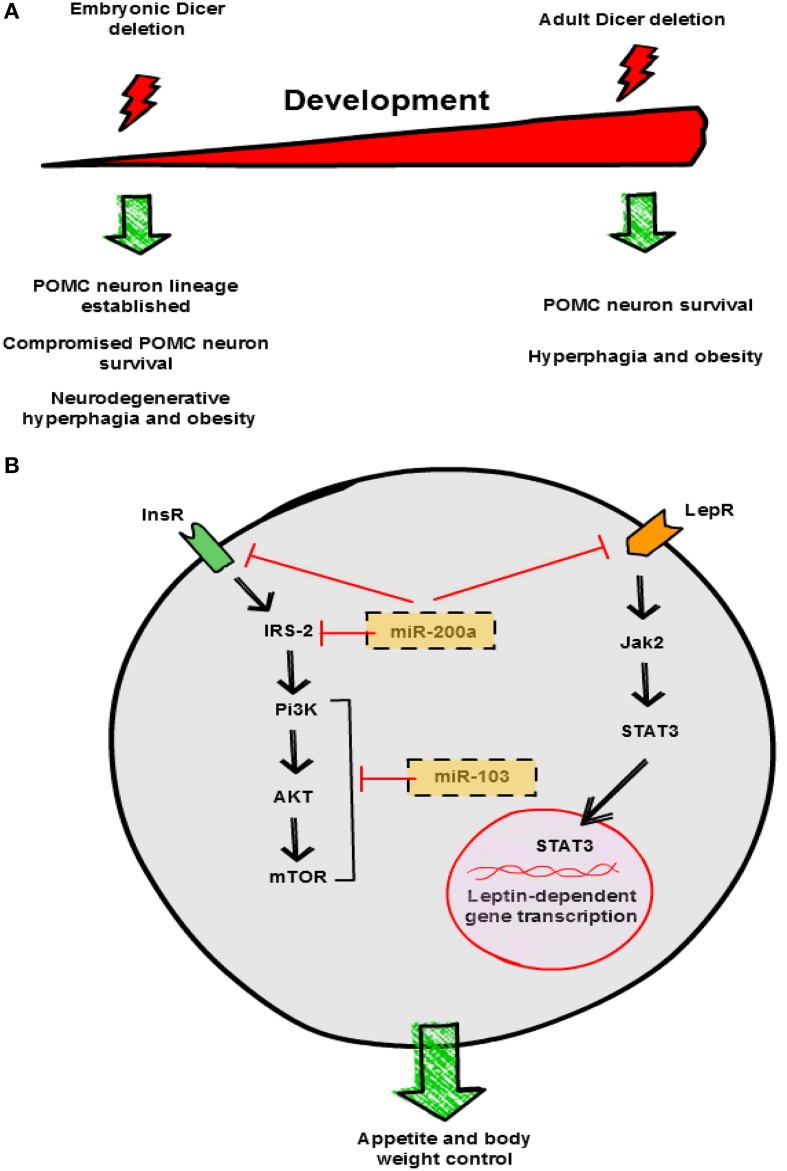
**Hypothalamic miRNAs control energy balance**. **(A)** Divergent effects of Dicer deletion on survival of hypothalamic neurons. Loss of Dicer in POMC neurons during embryonic development does not interfere with neuronal lineage establishment but leads to post-natal neurodegeneration. In contrast, lack of Dicer in the adult does not interfere with POMC neuron viability. Both experimental manipulations cause hyperphagia and obesity in mice, although the underlying mechanisms are different. **(B)** Proposed mechanisms of action of miR-200a and miR-103 in neuronal insulin and leptin signaling pathways. This graphical summary is based on reports by Schneeberger et al. (2012), Greenman et al. (2013), Crepin et al. (2014) and Vinnikov et al. (2014). InsR, insulin receptor; LepR, leptin receptor.

In the hypothalamus, *Dicer* transcript expression is regulated by nutrient availability, pathophysiological conditions of nutrient excess and genetic obesity. Fasting up-regulates *Dicer*, while the expression of other transcripts implicated in miRNA biogenesis were unaltered. In contrast, genetic and induced rodent models of energy excess exhibited decreased expression of *Dicer* in the hypothalamus (Schneeberger et al., 2012). These results suggested, for the first time, a potential physiological role for Dicer and miRNA biogenesis in the regulation of systemic energy homeostasis. Consistent with this, adult deletion of Dicer in the ARC caused hyperphagia and obesity, a phenotype that was not the consequence of neuronal cell death (Figure 2A) (Vinnikov et al., 2014).

Dicer is expressed in virtually all POMC and AgRP neurons of the ARC. Conditional loss of Dicer in POMC neurons leads to obesity from 6 weeks of age onwards (Schneeberger et al., 2012; Greenman et al., 2013). Dicer-dependent miRNAs seem to be essential for POMC neuron survival, as progressive loss of POMC neurons was reported in two studies (Figure 2A) (Schneeberger et al., 2012; Greenman et al., 2013). Similar to other neurodegenerative models (Xu et al., 2005; Ryu et al., 2008; Susaki et al., 2010), attenuated anorexigenic tone is likely the cause of the obese phenotype in POMC Dicer KO mice. Interestingly, a global transcriptomic approach showed alterations in specific signaling pathways associated to classical neurodegenerative disorders, suggesting similar underlying mechanisms (Schneeberger et al., 2012). Collectively, these results indicate that Dicer-dependent miRNAs are essential for post-natal POMC neuron survival and maintenance but are dispensable for POMC neuron lineage establishment (Figure 2A).

## Regional-specific miRNAs expressed in the hypothalamus

It is well documented that each tissue exhibits a particular miRNA expression profile, suggesting specific functions. In this regard, initial studies reported that the rodent hypothalamus is differentially enriched in specific miRNAs such as miR-124a, miR-125a, miR-136, miR-138, miR-212, miR-338, miR-451, let-7c genes and particularly miR-7a and miR-7b (Farh et al., 2005; Bak et al., 2008). These results have been recently confirmed and expanded using high-throughput sequencing. miRNA expression profiling of the ARC and paraventricular nucleus (PVN) of the rat hypothalamus has revealed similar expression patterns from a set of >210 miRNA genes (Amar et al., 2012). A prototype profile for these two hypothalamic nuclei is defined by ~20 miRNAs, including seven of the eight genes of the let-7 family, miR-9, miR-30, and the two miR-7 genes (Amar et al., 2012).

Consistently, *in situ* hybridization studies aimed at establishing the cellular localization of miR-7a and miR-7b, showed a more restricted expression pattern for the former being mainly located in the ARC (Herzer et al., 2012). Specifically, miR-7a is preferentially expressed in AgRP neurons rather than POMC neurons (Herzer et al., 2012). These studies indicate the existence of hypothalamic-enriched miRNAs suggesting specific pathophysiological functions for miRNAs upon energy balance control.

## Functions of specific miRNAs in the hypothalamic regulation of energy homeostasis

The identity of particular hypothalamic miRNAs implicated in systemic energy balance control just started to be unveiled. In an attempt to describe relevant hypothalamic miRNAs, recent studies have profiled miRNA expression in the context of metabolic distress. Crepin et al., investigated differential miRNA expression patterns in the hypothalamus from leptin-deficient *ob/ob* mice versus controls using Taqman microarray technology (Crepin et al., 2014). They found 11 out of 524 miRNAs significantly modified with a fold-change >2 in *ob/ob* mice: 10 were up-regulated and 1 down-regulated. Increased expression was confirmed for only 3 miRNAs (miR-200a, miR-200b and miR-429), and their expression was normalized after leptin treatment. miR-200a was also increased in the hypothalamus of leptin receptor deficient *db/db* mice (Crepin et al., 2014). *In silico* and luciferase activity studies indicated that Insulin receptor 2 (Irs-2) and leptin receptor are direct targets of miR-200a (Figure 2B), and intracerebroventricular (i.c.v.) blockade of miR-200a ameliorated the metabolic alterations of *ob/ob* mice and normalized the expression of these genes (Crepin et al., 2014). In a previous study, using a similar large-scale approach, these authors reported that impairment of leptin action perinatally also caused disturbances in hypothalamic miRNAs expression. Administration of a leptin antagonist in newborn rats promoted overweight and leptin/insulin resistance as well as changes in hypothalamic miRNA expression profile in adulthood (Benoit et al., 2013). Interestingly, 38 miRNAs were found to be differentially expressed including miR-200a (Benoit et al., 2013). Together, these results indicate that overexpression of miR-200a in obesity may interfere with insulin and leptin pathways in the hypothalamus by down-regulating key signaling mediators such as Irs-2 and leptin receptor respectively (Figure 2B).

Another study assessed the effects of short and long-term nutritional manipulations on hypothalamic miRNA expression in rats (Sangiao-Alvarellos et al., 2014). Chronic calorie restriction (CR) or high-fat diet (HFD) administration altered the expression of 74 out of 641 miRNAs analyzed, including let-7a, miR-9^*^, miR-30e, miR-132, miR-145, miR-200a, and miR-218 (Sangiao-Alvarellos et al., 2014). Algorithm-based target predictions included key components of signaling pathways found to be disturbed in obesity, such as NF-κβ, interleukins, PI3K/AKT, ceramides, insulin receptor, p70S6K and JAK/STAT (Sangiao-Alvarellos et al., 2014).

miR-103 has also been recently implicated in the hypothalamic control of energy homeostasis. Using a combination of hypothalamic miRNA profiling and *in silico* predictions, Vinnikov and collaborators found that bilateral injection of miR-103 mimic attenuated the obesogenic phenotype of mice lacking Dicer in forebrain neurons (Vinnikov et al., 2014). This effect was likely mediated by the ability of miR-103 to modulate hypothalamic PI3K-Akt-mTOR activity, key components of the insulin signaling pathway, although the precise target remains unknown (Figure 2B) (Vinnikov et al., 2014).

## Conclusions and future directions

Hypothalamic expression of miRNA processing machinery genes and specific miRNAs are modulated by nutrient availability, pathophysiological conditions of nutrient excess and genetic obesity. These findings strongly posit miRNAs as physiological candidates implicated in the hypothalamic control of systemic energy balance. Despite these exciting observations, the precise roles of specific miRNAs in the hypothalamus upon regulation of appetite, energy expenditure and metabolism remain largely unknown. Few studies have started to address this question and indentified a battery of miRNAs that are regulated by nutritional/metabolic manipulations. To date only miR-200a and miR-103 have been causally implicated in the hypothalamic control of whole-body energy homeostasis, although many additional miRNAs may be involved in the modulation of this complex biological process. Future studies will need to identify which specific sets of miRNAs are expressed in a neuronal type-specific manner and how they correlate with expression of target genes and physiological outputs. This specificity is particularly important, as the hypothalamus contains a number of different neuronal types with divergent functions. Furthermore, the generation of conditional miRNA KO mice will provide valuable insights into the function of specific miRNAs in particular subpopulations of hypothalamic neurons upon energy balance and metabolism. The advent of innovative genetically-engineered mouse lines in combination with high-throughput sequencing and omics approaches will provide novel and unanticipated insights into fundamental aspects of hypothalamic network biology and energy homeostasis regulation.

### Conflict of interest statement

The authors declare that the research was conducted in the absence of any commercial or financial relationships that could be construed as a potential conflict of interest.
