# Corrigendum: Hypothalamic miRNAs: emerging roles in energy balance control

**DOI:** 10.3389/fnins.2015.00096

**Published:** 2015-03-20

**Authors:** Marc Schneeberger, Alicia G. Gómez-Valadés, Sara Ramírez, Ramon Gomis, Marc Claret

**Affiliations:** ^1^Diabetes and Obesity Research Laboratory, Institut d'Investigacions Biomèdiques August Pi i SunyerBarcelona, Spain; ^2^Endocrinology and Nutrition, Hospital Clínic - University of BarcelonaBarcelona, Spain; ^3^CIBER de Diabetes y Enfermedades Metabólicas AsociadasBarcelona, Spain

**Keywords:** miRNA, Hypothalamus, obesity, diabetes mellitus, POMC neurons

The acknowledgments section for this mini-review article was accidentally omitted.

## Acknowledgments

This work was supported by Grants PI13/01604, PI10/01074 from National R+D+I (Ministerio de Ciencia e Innovación; MICINN) cofunded by Instituto Salud Carlos III (ISCIII) and the ERDF (MC). Marie Curie People Cofund Fellowship, Seventh Framework Programme of the European Commission grant 267248:DIATRAIN (AGG-V). MS is a recipient of an undergraduate grant from the University of Barcelona. MC is a recipient of a Miguel Servet contract (MICINN-ISCIII, CP09/00233). This work was carried out in part at the Esther Koplowitz Centre.

### Conflict of interest statement

The authors declare that the research was conducted in the absence of any commercial or financial relationships that could be construed as a potential conflict of interest.

